# Evolution and adaptation of dengue virus in response to high-temperature passaging in mosquito cells

**DOI:** 10.1093/ve/veaf016

**Published:** 2025-04-24

**Authors:** Fhallon Ware-Gilmore, Matthew J Jones, Austin J Mejia, Nina L Dennington, Michelle D Audsley, Matthew D Hall, Carla M Sgrò, Theresa Buckley, Ganesh S Anand, Joyce Jose, Elizabeth A McGraw

**Affiliations:** Department of Entomology, The Pennsylvania State University, University Park, PA 16802, USA; The Center for Infectious Disease Dynamics, The Pennsylvania State University, University Park, PA 16802, USA; Department of Entomology, The Pennsylvania State University, University Park, PA 16802, USA; Department of Biology, The Pennsylvania State University, University Park, PA 16802, USA; Department of Entomology, The Pennsylvania State University, University Park, PA 16802, USA; The Center for Infectious Disease Dynamics, The Pennsylvania State University, University Park, PA 16802, USA; Department of Entomology, The Pennsylvania State University, University Park, PA 16802, USA; School of Biological Sciences, Monash University, Melbourne, VIC 3800, Australia; School of Biological Sciences, Monash University, Melbourne, VIC 3800, Australia; School of Biological Sciences, Monash University, Melbourne, VIC 3800, Australia; The Center for Infectious Disease Dynamics, The Pennsylvania State University, University Park, PA 16802, USA; Department of Chemistry, The Pennsylvania State University, University Park, PA 16802, USA; The Center for Infectious Disease Dynamics, The Pennsylvania State University, University Park, PA 16802, USA; Department of Chemistry, The Pennsylvania State University, University Park, PA 16802, USA; The Center for Infectious Disease Dynamics, The Pennsylvania State University, University Park, PA 16802, USA; Department of Biochemistry and Molecular Biology, The Pennsylvania State University, University Park, PA 16802, USA; Department of Entomology, The Pennsylvania State University, University Park, PA 16802, USA; Department of Biology, The Pennsylvania State University, University Park, PA 16802, USA

**Keywords:** Dengue, Virus Evolution, Mosquito Vector, Adaptation, Climate Change

## Abstract

The incidence of arboviral diseases like dengue, chikungunya, and yellow fever continues to rise in association with the expanding geographic ranges of their vectors, *Aedes aegypti* and *Aedes albopictus*. The distribution of these vectors is believed to be driven in part by climate change and increasing urbanization. Arboviruses navigate a wide range of temperatures as they transition from ectothermic vectors (from 15°C to 35°C) to humans (37°C) and back again, but the role that temperature plays in driving the evolution of arboviruses remains largely unknown. Here, we passaged replicate dengue serotype-2 virus populations 10 times at either 26°C (Low) or 37°C (High) in C6/36 *Aedes albopictus* cells to explore the differences in adaptation to these thermal environments. We then deep-sequenced the resulting passaged dengue virus populations and tested their replicative fitness in an all-cross temperature regime. We also assessed the ability of the passaged viruses to replicate in the insect vector. While viruses from both thermal regimes accumulated substitutions, only those reared in the 37°C treatments exhibited nonsynonymous changes, including several in the E, or envelope protein, and multiple non-structural genes. Passaging at the higher temperature also led to reduced replicative ability at 26°C in both cells and mosquitoes. One of the mutations in the E gene involved the loss of a glycosylation site previously shown to reduce infectivity in the vector. These findings suggest that viruses selected for growth at higher ambient temperatures may experience tradeoffs between thermostability and replication in the vector. Such associations might also have implications for the suitability of virus transmission under a changing climate.

## 1. Introduction

Mosquito-borne viruses and climate change are two threats that have converged to affect global public health (Reiter 2001, Gould and Higgs 2009, Halsch et al. 2021). Climate change, including increases in global mean temperatures and extreme climatic events, such as heatwaves, droughts, and increased rainfall, can affect the geographic distributions of mosquito species and the pathogens they transmit (Lafferty 2009, Parham et al. 2015). Temperature influences mosquito and pathogen life history traits, including larval and adult survival, development, fecundity, and the rates of pathogen replication (Alto and Bettinardi 2013, Carrington et al. 2013, Mordecai et al. 2019). With changes in global climate, it is expected that modifications in the performance of these traits may contribute to shifts in disease risk across a fitness landscape (Fan et al. 2015, Mordecai et al. 2019, Cator et al. 2020).

An emerging body of literature investigating the relationship between mosquito-borne disease and climate change through experimental and modeling studies has mapped the likely expected future distributions of common vectors and pathogens (Mordecai et al. 2017, Ryan et al. 2019, Dennington et al. 2024). Such studies suggest an increase in prevalence in some regions for specific vector-borne pathogens [e.g. dengue (DENV), chikungunya (CHIKV), West Nile Virus (WNV)], and net decreases and geographical shifts in optimal habitats for others, including malaria (Lafferty 2009, Escobar et al. 2016, Murdock et al. 2016, Ryan et al. 2019, 2021, Mordecai et al. 2020, Shocket et al. 2020). Of the arboviruses, DENV (*Flaviviridae*, Flavivirus) and its four serotypes have the broadest geographic range, transmitted by its primary arthropod vector, *Aedes aegypti*, a highly anthropophilic mosquito species adapted to both tropical and subtropical latitudes (Gubler 1998, 2002). With few exceptions (Dennington et al. 2024), predictive models for vector-borne diseases do not incorporate how pathogen and vector adaptation to a changing climate may affect geographic distributions. Particularly for viruses, more experimental work is needed to ascertain how temperature governs virus fitness in a changing landscape (Ciota and Kramer 2010, Coffey et al. 2013). Recent outbreaks of chikungunya fever (Cunha et al. 2020) and the resurgence of dengue fever in South America, underline the urgency in addressing this knowledge gap (Ebi and Nealon, 2016; Gould and Higgs 2009).

Arboviruses, including DENV, necessarily cycle between two different hosts (i.e. vertebrate host and blood-feeding arthropod) (Whitehorn and Yacoub 2019) and are, therefore, evolutionarily constrained by these distinct biological contexts (Bellone and Failloux, 2020, Coffey et al. 2013). Examples of factors known to differ between vectors and mammals include temperature (Bellone and Failloux, 2020, Kinney et al. 2006, Bisht and Te Velthuis 2022), host cell receptors (Trammell et al. 2021), antiviral immune responses (Trammell et al. 2021), cell types (Cappuccio and Maisse 2020), and tissue barriers (Franz et al. 2015). In their most common vertebrate host, humans, DENV can replicate at temperatures ranging from 37°C to 44°C (Kinney et al. 2006). Once consumed by a blood-feeding mosquito, commonly *Aedes species*, they switch to replicating in their invertebrate host through replication within the midgut and later the salivary glands, whose internal thermal environment depends on the ambient temperature and the insect’s behavior (Huang et al. 2019). The thermal performance range for adult *A. aegypti* is 15–32°C, with an optimum <27°C (Reinhold et al. 2018). Higher temperatures tend to be associated with faster virus replication and a greater mutation rate (Corner and Ng 1987). The temperature of the host or vector can also affect lipid, nucleic acid, and protein structure and functions and is, therefore, a likely key factor affecting the virus’s interactions with cellular components during replication (Ciota and Kramer 2010, Perera et al. 2012, Scaturro et al. 2015).

Research regarding the role of environmental variation, specifically temperature, in shaping arbovirus evolution is limited (Bellone and Failloux, 2020, Fay et al. 2021). Viruses rely on a significant amount of plasticity and high genetic variation to be successful across disparate host environments (Weaver 2006). The high mutation rate of RNA viruses (10^−3^ to 10^−5^ errors/site/replication), for example, resulting from an error-prone RNA polymerase and the short replication times, can drive rapid evolution. This variation is key to Flavivirus adaptability and the potential for rapid fitness gains (Ciota et al. 2008, 2012, Grubaugh and Ebel 2016, Ehrbar et al. 2017). In this study, we examine the potential for a strain of DENV serotype-2, with a global distribution, to evolve in response to consistent passaging at either a vector (26°C) or host-relevant temperature (37°C) in cell culture. We then measured *in vitro* replication rates and *in vivo* infectivity of the resulting passaged viruses across the low and high-temperature environments, relating these infection phenotypes with evolved genetic changes in the virus.

## 2. Materials and methods

### 2.1 Virus culture

DENV-2 (ET300 strain) was passaged at two temperatures, 26°C and 37°C in healthy, established *Aedes albopictus* C6/36 mosquito cells absent of *Wolbachia* ET300 (Genbank EF440433.1), was isolated in Australia in 2016 from a patient with a travel history in East Timor. *Aedes albopictus* C6/36 cells were grown at 26°C in RPMI 1640 medium (Invitrogen, Carlsbad, CA, USA) supplemented with 10% fetal bovine serum (FBS), 1× Glutamax (Invitrogen), and HEPES buffer. Cells were first allowed to form monolayers of ∼60–80% confluence in T175 flasks (Sigma Aldrich, St. Louis, MO, USA) and then inoculated with frozen stock DENV (MOI of 1.0) and maintained in RPMI medium supplemented with 2% FBS. At 7 days postinoculation, cell medium was collected from the flask, and pooled virus was quantified via qRT-PCR. The final pooled virus concentration was 10^6^ genome copies per milliliter. This ancestral passage of the virus (passaged 0 times) was stored at −80°C in single aliquots for the seeding of the passaging regime and as a control for the replicative fitness assays conducted after virus evolution.

### 2.2 Virus passaging/selection regime

**Figure 1.**
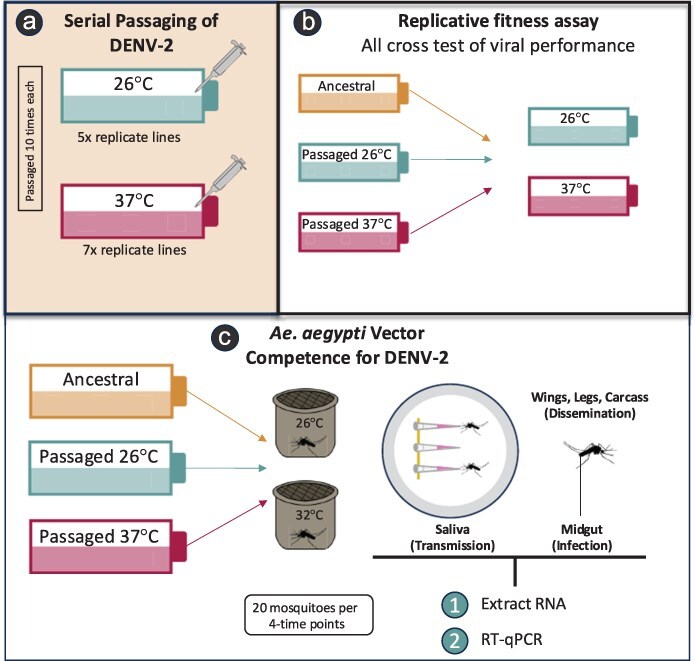
**Study design workflow. (A)** DENV-2 (ET300) was passaged at two temperatures in *Aedes albopictus* C6/36 mosquito cells at seven days post-inoculation. Cell medium was collected, pooled, and virus was quantified via qRT-PCR, resulting in 10^6^ genome copies per ml. This virus was used for sequential passaging and was a control for the replicative fitness assays conducted after virus evolution. During the selection regime, lines were passaged every three days, and in total, there were 12 replicate lines, 26°C n=5 and 37°C n=7, each passaged for a total of 10 generations. At each passage, 500 µl of viral supernatant was transferred to a new set of uninfected cells. **(B)** A replicative fitness assay was conducted after passaging to test for phenotypic change. Replication kinetics of the ancestral and passaged virus were assessed at alternate temperatures in an all-cross design. **(C)** To determine vector competence, *Ae. aegypti* mosquitoes aged seven days were fed either ancestral or passaged virus. Blood fed mosquitoes were randomly sorted into gallon cartons and held either at 26°C or 32°C. 32°C was selected instead of 37°C because mosquito mortality is very high under the latter. Infection (IR), dissemination (DR), and transmission rates (TR) were assessed for the relevant tissues at 3,6,9,12 dpi. Mosquitoes were anesthetized on ice in cohorts of 20, and saliva was collected by inserting the proboscis in a pipette tip containing a salivation solution, which allowed them to salivate for 30 minutes. Midgut and carcass (containing wings and legs) were collected in separate tubes containing Trizol (Invitrogen, Waltham, Massachusetts, USA). Viral RNA was extracted, and DENV titers were determined for each tissue using qRT-PCR.

DENV-2 was passaged at 26°C and 37°C, representing the low- and high-temperature regimes, respectively (Fig. 1), and the optimal adult *A. aegypti* (Reinhold et al. 2018) and human body temperatures, respectively. Lines were passaged every 3 days, and in total, there were 12 replicate lines, 26°C, *n* = 5, and 37°C, *n* = 7, each passaged 10 times. At each passage, 500 µl of viral supernatant was transferred to a new set of uninfected cells that were grown at 26°C (not pre-adapted) before they were transferred to their treatment temperature. The remaining supernatant was stored in individual aliquots for subsequent viral sequencing at −80°C, while additional samples were stored in liquid nitrogen for restarting viral cultures for replicative fitness assays as needed post-selection. Daily sub-samples were collected during passages for rapid viral load quantification after a simple extraction. In brief, 20 μl of supernatant was mixed 1:1 with extraction butter comprised of 10 mM Tris buffer, 1 mM EDTA, 50 mM NaCl, 0.25 μl proteinase K (Roche#3 115 828 001). Samples were incubated at 56°C for 5 min and at 98°C for an additional 5 min and immediately used for virus quantification (see Section 2.5).

### 2.3 DENV quantification

DENV copies were determined using the TaqMan fast virus 1-step Master Mix with a final volume of 10 μl encompassing DENV primers and probe (500 nM per reaction) and 2.5 μl of extracted RNA. Primer and probe sequences for the detection of DENV (Supplementary Table 1) and the construction of standards were as previously described (Ye et al. 2014). In brief, the PCR-amplified fragment was inserted into a plasmid, which was transformed into *Escherichia coli*. The final fragment was then serially diluted, ranging from 10^7^ to 10 copies/reaction of DENV-2 fragment copies, which were used to create a standard curve of DENV amplification. Samples were run in duplicate on 96-well plates (Life Technologies, Carlsbad, CA, USA) and sealed with MicroAmp™optical adhesive film. DENV concentrations were expressed as copies per microliter of viral supernatant, and the standard curves were each run-in duplicate on 96-well plates with the limit of detection set at 10^2^ copies. Thermal cycling conditions were run on the LightCycler 480 (Roche Applied Science, Switzerland) as follows: 50°C for 5 min for reverse transcription following 95°C for 20 min, amplification cycling at 95°C for 3 s, and 60°C for 30 s.

### 2.4 *In vitro* replicative fitness assay

After 10 passages, each of the 12 (26**°**C treatments, *n* = 5, in 37**°**C, *n* = 7) replicated passaged lines was assayed for their replicative fitness in both the original passage temperature as well as the alternate in an all-cross design. In addition to the passaged lines, we also included the ancestral virus used to seed the initial passages. The history of passage x×temperature treatments were, therefore, as follows: Ancestral tested at 26**°**C, Ancestral tested at 37**°**C, 26**°**C passaged tested at 26**°**C, 26**°**C passaged tested at 37**°**C, 37**°**C passaged tested at 26**°**C, and 37**°**C passaged tested at 37**°**C. The treatments were infected as previously described in Section 2.1, with a multiplicity of infection of 1.0. After 3 days postinoculation of passaged virus into a flask, 5 µl of viral supernatant was sampled three times a day (7:00 am, 3:00 pm, 11:00 pm) for 8 days to capture virus growth rates. Viral loads were quantified as above via simple extraction and qRT-PCR.

### 2.5 Sequencing of passaged viruses

To assess any genetic changes that occurred during the passaging regime, viral supernatant was collected from all flasks postselection and extracted via Trizol LS reagent (Invitrogen, Carlsbad, CA, USA) according to the manufacturer’s protocol. This was followed with RNA purification using the Qiagen RNeasy kit, in which the purified RNA was eluted into 30 µl of RNase-free water. Samples were then sent to Azenta (Plainfield, New Jersey, USA) for processing. In brief, RNA samples were quantified using a Qubit 2.0 Fluorometer (ThermoFisher Scientific, Waltham, MA, USA), and RNA integrity was checked with a 4200 TapeStation (Agilent Technologies, Palo Alto, CA, USA). Samples were then treated with TURBO DNase (Thermo Fisher Scientific, Waltham, MA, USA) as per the manufacturer’s protocol. An rRNA depletion sequencing library was prepared using QIAGEN FastSelect rRNA HMR Kit (Qiagen, Hilden, Germany) and the NEBNext Ultra II RNA Library Preparation Kit for Illumina (NEB, Ipswich, MA, USA). Sequencing libraries were validated and quantified as above, as well as by quantitative PCR (KAPA Biosystems, Wilmington, MA, USA). The sequencing libraries were multiplexed and clustered on a single flow cell. After clustering, the flow cell was loaded on the Illumina HiSeq instrument according to the manufacturer’s instructions. The samples were sequenced using a 2 × 150 Pair-End (PE) configuration. Raw sequence data (.bcl files) generated from Illumina HiSeq were converted into fastq files and de-multiplexed using Illumina bcl2fastq program version 2.20. One mismatch was allowed for index sequence identification. After demultiplexing, sequence data were checked for overall quality and yield. Then, raw sequence reads were trimmed to remove possible adapter sequences and nucleotides with poor quality using Trimmomatic v.0.36 (Bolger et al. 2014). The reads were then mapped to the Dengue virus type 2 strain TSV01 reference genome (AY037116) available on NCBI using bwa v.0.7.12 (Li and Durbin 2009). SNPs/INDELs were detected using SAMtools mpileup (Li et al. 2009) in conjunction with VarScan v.2.3.9 (Koboldt et al. 2009). The settings of VarScan were minimum coverage: 10, minimum reads: 4, minimum variant frequency: 0.5%, minimum *P*-value: .05. Comparisons were made between each evolved line/replicate, and the ancestral and nonsynonymous substitutions in coding regions following nucleotide alignment using Clustal Omega (Sievers et al. 2011). All differences between lines were then visually confirmed in the original sequence data using NCBI Genome Data Viewer (Rangwala et al. 2021).

### 2.6 Mosquito culture

*Aedes aegypti* eggs, originally collected from Mérida, Mexico by Pablo Manrique Saide (Universidad Autonoma de Yucatan) and obtained via Zhiyong Xi (Michigan State University), were hatched in 1 liter of reverse osmosis (RO) autoclaved water containing 500 ml of used larval water from the previous generation. Second instar larvae were spread into trays of no more than 250–300 individuals to ensure low-density rearing in 3 l of RO water. Larvae were fed Tetramin fish food (Melle, Germany). Upon emergence, adults from each pan above were reared in 45 cm square cages (BioQuip) and fed 10% sucrose ad libitum. All stages were reared under standard conditions: 12  h light/dark, 26°C, 60% relative humidity.

### 2.7 *In vivo* infectivity of passaged lines

Given the scale of vector competence work, we selected a single-virus strain postpassaging, representing the 26**°**C and 37**°**C treatments, to test for replication in the vector. We also included the ancestral virus. Vector competence methods are as previously reported (Terradas et al. 2017). In brief, 7-day-old female mosquitoes were sorted into groups of 80–90 in 68 oz 21 in × 17 in × 14 in paper soup cartons with mesh coverings. Sucrose, as fed by soaked cotton, was removed 24 h before the feed and replaced with water to encourage blood feeding. An artificial blood feeder (Hemotek) was used to deliver blood meals through pig intestines that had previously been soaked in 10% sucrose solution for 24 h. A 1:1 mix of defibrinated human blood (BioIVT, Westbury, New York) and either ancestral or passaged viruses revived from the freezer and freshly grown were placed inside the feeder. The final fed concentration of each virus was 2.2e^6^ DENV copies/ml. After the infectious feed, all mosquitoes were visually inspected under chilling to remove any unfed mosquitoes. The remaining individuals were sorted into 40-oz paper cartons, each containing 30 female mosquitoes. Mosquitoes were held until their collection day with continuous access to 10% sucrose.

We collected twenty individuals per line at 3-, 6-, 9-, and 12 days post-infection (dpi). We dissected their midguts, carcass, and saliva, representing their infection rate (IR), dissemination rate (DR), and their transmission (TR), respectively. We report prevalence (% infected) and DENV viral loads for all infected individuals. To carry out the dissections, mosquitoes were anesthetized with triethylamine (Sigma, St. Louis, MO, USA) to induce paralysis and kept on ice. To induce salivation, each mosquito’s proboscis was inserted into a sterile 200 μl pipette tip containing the following solution: 15 µl of 1 mM ATP, FBS, and 30% sucrose as previously reported (Dutra et al. 2016). Mosquitoes were allowed to salivate for 30 min, and during the salivation period, mosquitoes were visually inspected to ensure survival by checking for movement. The collected contents in the pipette tips were expelled into a 1.5 ml microfuge tubes. The mosquito midgut and “carcass” (remainder including legs and wings) were placed in separate 1.5 ml tubes containing 300 µl of Trizol (Invitrogen, Waltham, MA, USA) and a 2 mm diameter glass bead (Merck KGaA, Darmstadt, Germany). Tissues were homogenized using the Bead Ruptor Elite bead mill (Biospec Products, Bartlesville, OK, USA). All samples were stored at −80°C. Samples were subsequently extracted following the manufacturer’s protocol. Virus was then quantified as above using qRT-PCR.

### 2.8 Protein structure analysis

Amino acid changes in E protein were analyzed using the highest resolution cryoEM structure of DENV Binjari virus PDB ID: 7KV8. The molecular graphics and analyses were generated using ChimeraX (Resource for Biocomputing, Visualization, and Informatics at the University of California, San Francisco, Office of Cyber Infrastructure and Computational Biology, USA) (Pettersen et al. 2021).

## 3. Results

### 3.1 Viral loads during serial passaging

**Figure 2.**
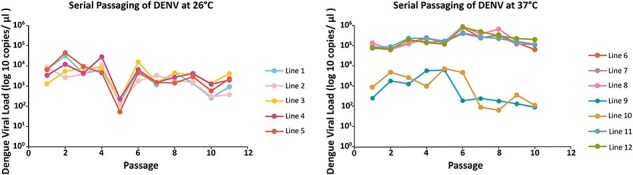
Viral loads during serial passaging of DENV-2 at a high and low temperature in C6/36 cells. Sequential passaging of an Ancestral DENV isolate in C6/36 mosquito cells at 26 °C (5 replicate lines) and 37°C (7 replicate lines). Individual points denote Log _10_ transformed viral genome copies for each line at every passage. Virus was passaged every three days, and each day virus was collected to access replication dynamics. Each point is the highest load for a 24-hour period across days per passage. Grand mean across replicate lines and samples are shown in Supplementary Figure 1.

During serial passaging, we sampled multiple times per day and across all passages to explore viral replication under the two temperatures (26°C and 37°C). These temperatures were selected to represent the vector and host environments. Viral loads varied by both passage number (ANOVA, DF = 10, *F*stat = 9.88, *P* < .002) and temperature (ANOVA, DF = 1, *F*stat = 171.625, *P* < .001). DENV-2 reared at the lower temperature (Fig. 2a) not surprisingly grew to lower overall loads than 37°C (Fig. 2b), but also exhibited more significant variation. The average daily peak viral load at 26°C was 10^3^ genome copies per microliter, while those at 37°C were 10^4^ times genome copies per microliter, ∼10-fold higher. Our difference in the number of replicates, 5 vs 7 for 26°C and 37°C, respectively, was based on the concern that we may be more likely to lose lines reared at the higher temperature due to overgrowth. Two of the 37°C lines (replicates 9 and 10) did struggle, reaching as low as 10^1^ genome copies per microliter at passage 5.

### 3.2 Assessment of temperature-specific effects of experimental passaging on replicative fitness

**Figure 3.**
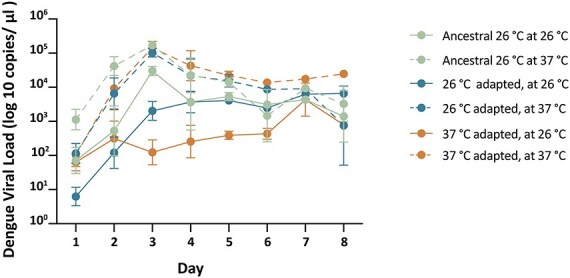
Daily average viral load from replicative fitness assays. Viral performance at the two temperatures following the passaging regime. Load represented as means and SEM with each point representing the mean of three individual collections per day (7:00AM, 1:00PM, 11:00PM) averaged across line replicates (all replicate lines can be found in Supplementary figure 2). There was a significant effect of passaging temperature (“Treatment” F = 40.96, df = 5, p <.0001) on replicative fitness, which was observed to vary by day (“Day”: F = 81.24, df = 5, p <.0001). Higher loads were seen for lines passaged at 37°C and tested at 26°C, compared to those passaged at 26°C. Only the 37°C passaged lines exhibited reduced fitness when tested at 26°C post-passaging.

To evaluate if there were fitness gains due to the serial passaging regime (Fig. 1a), we assessed the replicative fitness of all lines from Passage 10 (P_10_) in both the native and novel temperature compared to passaging (Fig. 1b). For comparison, we also included the ancestral line. All inoculations were standardized at 2.5 × 10^5^ virus copies per microliters of supernatant. The supernatant was collected and tested for viral loads at three time points each day (7 a.m., 1 p.m., and 11 p.m.) over 8 days postinoculation (Fig. 3). Using linear regression, we tested the effect of “treatment” and “day” on viral isolates.

We saw a significant effect of passaging treatment (*F* = 40.96, df  = 5, *P* <.0001) on replicative fitness, that varied by day (*F* = 81.24, df = 5, *P* < .0001). Not surprisingly, Tukey’s post-hoc comparisons (Supplementary Table 2) showed no difference in viral loads between the ancestral and 26°C passaged viruses when tested at 26°C (*P *= .62). Similarly, there was no difference between viral loads of the ancestral and 37°C passaged viruses when tested at 37°C *(P *= 1.00). For viruses passaged at 26°C and tested at 37°C, we saw (Fig. 3) higher replication rates (2- to 3-fold increase) when compared to the line that was passaged at 26°C and tested in the same environment (*P* < .0001) (Supplementary Table 3). This comparison simply demonstrates greater replication rates at higher temperatures, as seen in our passaging regime (Fig. 2). Replication rates between the Ancestral and 26°C passaged line both tested at 37°C showed no significant differences (*P *= .97), showing that the passaging at a lower temperature did not select for reduced fitness at the higher temperature. In contrast, virus passaged at 37°C and tested at 26°C, showed a decline in replicative ability compared to lines passaged at 26°C (*P *= .0016) and the Ancestral (*P *= .0030). Virus selected at 37°C and reared at 26°C performed the poorest compared to all other selection treatments and had a final viral load of 10^3^ viral genome copies per microliters of supernatant, suggesting an overall decrease in viral fitness resulting from passaging history (Fig. 3).

### 3.3. Genetic changes during passaging

Whole-genome sequencing of viruses from each replicate representing both temperature regimes at passage 10 was carried out to identify mutations arising during passaging. We sequenced the first five replicates of each treatment (lines 1–5 at 26°C and 6–10 at 37°C). Several genetic changes occurred in the passaged lines for each temperature treatment (Table 1) relative to the Ancestral line. The 26°C passaged lines experienced two common changes that were both synonymous changes and found in nonstructural protein genes (NS1, NS2A). Their presence across all five replicate lines suggested that they occurred convergently, or more likely, in the initial round of viral growth. For viruses selected at 37°C, passaging only produced nonsynonymous changes (6 in total) in structural and nonstructural protein-encoding genes, which could underpin the phenotypic shifts we saw in replicative fitness at 26°C. They might also explain poor replication of lines 9 and 10 at 37°C (Fig. 2); however, there may also be stochastic bottlenecks at work, given that some of these mutations are shared by lines that grew well (Table 1). Two were in the envelope (E) protein-encoding gene and present only in replicate 6. Many of the changes in the nonstructural encoding genes were convergent. In nonstructural protein 2A (NS2A), one change occurred in two out of five replicates. Two mutations in the nonstructural protein 2B (NS2B) appeared in three and four of the five replicates. A final change was identified in the nonstructural protein 4A (NS4A) region that was present in three out of five replicate lines. Four of the six nonsynonymous changes across three of the genes were from Isoleucine (I) to Leucine (L). To place these mutations in the context of natural genetic variation, we downloaded all 91 complete genomes of DENV serotype-2 available in GenBank (accessed 2 February 2025) (Sayers et al. 2022), aligning them with MEGA Evolutionary Genomics Software (Tamura et al. 2007). We note that very few of the mutations (Table 1) were present in the published genomes at all, or with any frequency.

**Table 1.** Location and nature of all nucleotide differences across the genome following 10 passages in *Aedes albopictus* (C6/36) cells relative to the Ancestral virus. We sequenced five lines representing each passaged treatment, numbered # 1-5 (26°C) and #6-10 (37°C). In all cases, mutations ocurred in ~99% of all sequence reads.

Genome position	Passage temperature	Gene (position)	AA site	Synonymous or nonsynonymous	Occurrence in replicate lineages
191	26°C	NS1(2613)	64	Synonymous	5/5 replicates (Lines 1, 2, 3,4, 5)
488	26°C	NS2A(3966)	163	Synonymous	5/5 replicates (Lines 1, 2, 3,4, 5)
1394	37°C	E (457)	153	Asparagine (N) > Serine (S)	1/5 replicates (Line 6) 0/91 DENV-2 genomes
2012	37°C	E (1075)	359	Isoleucine (I) > Threonine (T)	1/5 replicates (Line 6) 1/91 DENV-2 genomes
3884	37°C	NS2A (406)	136	Isoleucine (I) > Threonine (T)	2/5 replicates (Lines 6 and 9) 0/91 DENV-2 genomes
4471	37°C	NS2B (316)	106	Isoleucine (I) > Leucine (L)	4/5 replicates (Lines 6, 7, 8, 9) 0/91 DENV-2 genomes
4472	37°C	NS2B (317)	106	Isoleucine (I) > Threonine (T)	3/5 replicates (Lines 6, 7, 8, 9, 10) 3/91 DENV-2 genomes
6665	37°C	NS4A (279)	93	Tyrosine (Y) > Cysteine (C)	3/5 replicates (Lines 6, 7, 8) 0/91 DENV-2 genomes

**Figure 4.**
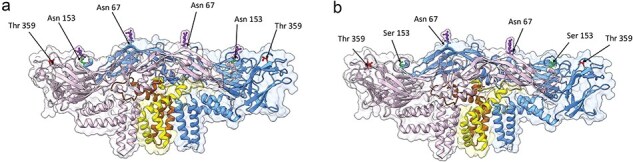
N153S mutation in E gene leads to the loss of glycosylation at N153. Surface-shaded side view of the dimeric structure of M and E heterodimers from the cryoEM structure of mature DENV-2 from PDB ID: 7KV8 is shown with E monomers (pink and blue); M monomers (yellow and brown). A) Wild-type virus structure showing the glycosylation sites on Asn 67 (green) and Asn 153 (green), with NAG in the ball and stick model (purple). Residue Thr 359 is indicated (red). B) The predicted structure of the N153S glycosylation site mutant was generated using ChimeraX. The glycosylation sites on Asn 67 (green) with NAG is shown in the ball and stick model (purple). The mutation of Ser 153 is indicated in green. Residue Thr 359 is indicated (red).

The E gene, N153S mutation, leads to the loss of glycosylation at N153. N153S is shown (Fig. 4) using the highest resolution cryoEM structure of DENV PDB ID: 7KV8 (Hardy et al., 2021). The figure shows the two glycosylation sites (N67, N153) in DENV E protein. Previous mutagenesis studies have demonstrated that the N153Q mutation eliminates a glycosylation site from DENV-2 E protein affecting DENV-2 replication in BHK and C6/36 cells but not in *A. aegypti* mosquitoes when intrathoracically injected (Bryant et al., 2007). A published structure (Fibriansah, et al. 2025) of the DENV-2 E protein glycan mutation shows the N153Q mutation in this structure abolishes the addition of *N*-acetyl-d-glucosamine (NAG) to the residue at 153, similar to what we have observed in this study, N153S. Analysis of this DENV structure revealed no significant changes in the E protein structure, confirming our observation that the removal of glycosylation site at 153 does not significantly affect the infectivity of DENV-2. We show the dimeric structures of the M–E heterodimers for visualizing the change in glycosylation (Fig. 4 and Supplementary Fig. 3). This observation allows us to conclude that the amino acid changes of E protein (N153S, I359T) did not result in a significant disruption of the overall protein structure compared to E protein from the wild-type virus. Interestingly, when the site is mutated in Zika virus, preventing glycosylation, the virus cannot infect the mosquito midgut after being introduced by oral feeding (Wen et al. 2018) Similarly, Zika mutants lacking this glycosylation, are much attenuated in mice exhibiting much reduced viremia (Fontes-Garfias et al. 2017).

Dengue virus nonstructural protein 2A (NS2A) is a replication protein with no known three-dimensional structure that has been shown to regulate flavivirus assembly (Xie et al. 2019). Based on the published topology of NS2A (Xie et al. 2013), the NS2A mutation I136 is located on transmembrane segment 5, presumably facing the ER lumen. I136 is likely interacting with structural prM, E, and NS1, which might contribute to producing infectious virions (Scaturro et al. 2015). NS2B protein acts as a cofactor for NS3, carrying out enzymatic reactions essential for viral replication, including protease and helicase activity. Although the crystallographic structure of a complete DENV NS3 molecule fused to 18 residues of the NS2B cofactor is known, the structure of the entire NS2B where the amino acid changes are observed is unknown (Luo et al. 2008). The NS2B mutations (I113L, I114T) are likely in the C-terminal region of NS2B, possibly interacting with the NS3 protease domain. Previously, a crystal structure of NS2B–NS3 has shown that a flexible loop interaction between NS2B and NS3 is required for conformational rearrangement of the NS2B, which is required for protease activation (Yildiz et al. 2013). Therefore, I113L and I114T are likely mediating the NS3 protease activity required for the polyprotein cleavage. Like NS2A, NS4A is a membrane protein that localizes on the ER membrane without structural information (Miller et al. 2007). Based on the predicted topology of NS4A, we predict that the Y93C mutation is located on a membrane-associated helix facing the ER lumen (Y. Li et al. 2018, Płaszczyca et al. 2019). Although the Y93 is not a conserved residue, Y93C is likely interacting with another protein via a disulfide bond or interacting with the ER membrane by cysteine modification.

### 3.4. *In vivo* virus performance

**Figure 5.**
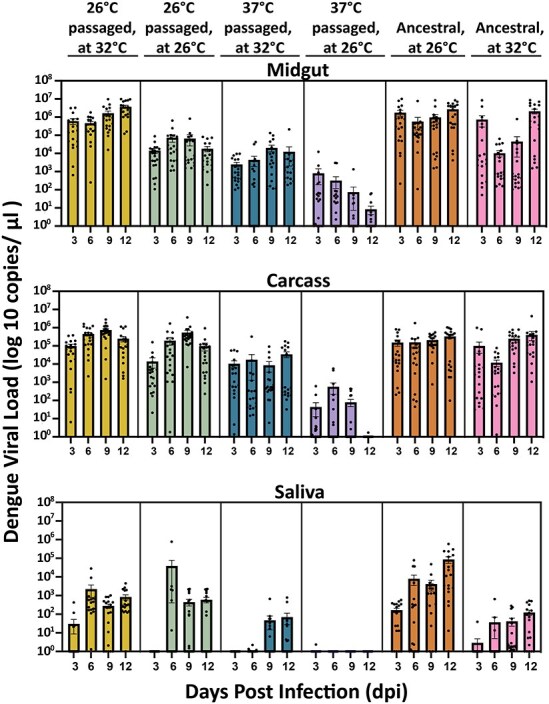
Viral load for Ae. aegypti mosquitoes following feeding with virus post-passage regime. Viral load for midgut (M), carcass (rest of the body), and saliva (S) after exposure to passaged and Ancestral DENV-2. Each point on the bar graph corresponds to a single mosquito, n=20 per treatment. Statistical significance between selection treatments is based on ANOVA (Supplementary Table 4) followed by post hoc multiple comparisons tests.

**Figure 6.**
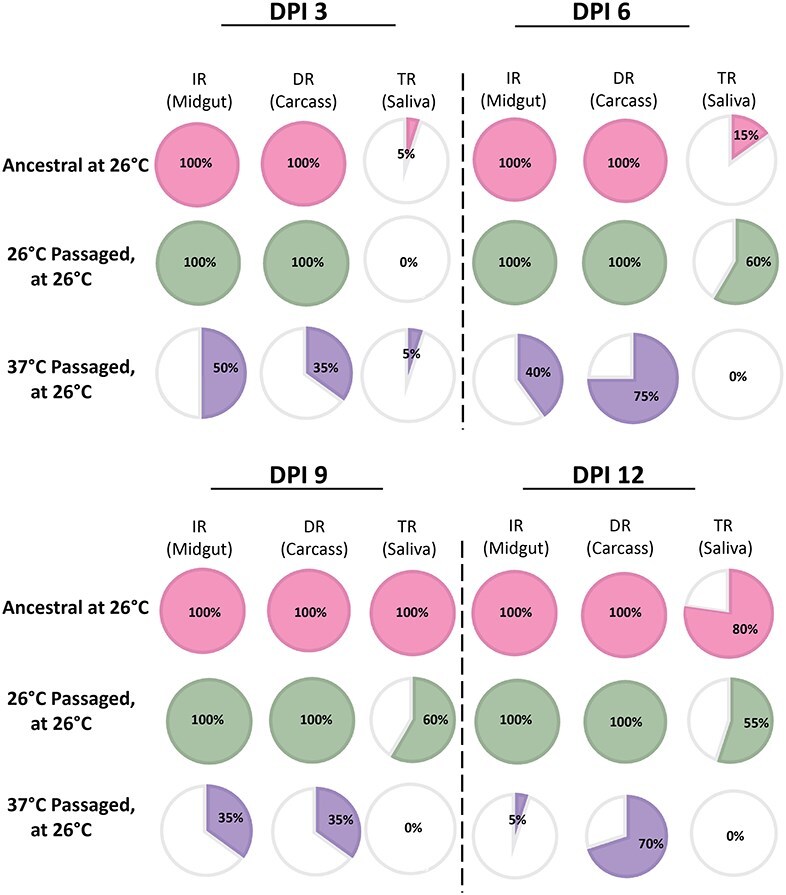
Infection prevalence for Ae. aegypti mosquitoes following feeding with virus post-passage regime. Pie charts comparing the prevalence of infection (IR), dissemination rate (DR) and transmission rate of challenged mosquitoes in selected treatments at DPI 3, 6, 9, 12 including: Ancestral at 26°C, 26°C passaged at 26°C, 37°C passaged at 26°C (Supplementary Table 5).

After viral passaging and genomic sequencing, we assessed whether the evolved lines exhibited any differences in vector competence, with mosquitoes being reared at two temperatures (26°C, 32°C). For these experiments, we selected a single representative lineage to test from each passaging temperature: line 1 (26°C) and line 6 (37°C) (Table 1). The latter was chosen because it contained all identified mutations. As mosquitoes were reared at 26°C, we expected that the 37°C passaged virus may also perform less well in the vector, as it did *in vitro* above. We examined both viral load (Fig. 5) and prevalence (Fig. 6) over a time course relevant to infection in the field, and in key tissues that would speak to the progression of the virus through the body and to the saliva (a proxy for transmission). We found that there was a significant relationship between viral load and virus treatment as per ANOVA for midgut (*P* < .0001), carcass (*P* < .0001), and saliva (*P* = .0047) (Supplementary Tables 4 and 5).

Mosquitoes challenged with Ancestral virus and reared at 26°C served as a control or baseline for comparison, infection was established in the midgut and disseminated to the rest of the body in all mosquitoes at all days postinfection (Fig. 6 and Supplementary Table 4; IR: 100%, DR: 100%). Infection prevalence was consistent across time and disseminated viral loads rose to 1 × 10^4^ viral copies per microliters of supernatant at 12 dpi (Fig. 5). Transmissibility trends differed across surveyed time points, with prevalence peaking at 9 dpi (TR: 100%) and decaying slightly at 12 dpi (TR: 75%) with 15/20 infected mosquitoes showing infected saliva (Supplementary Table 4). The post-hoc comparison indicates that there was no significant difference for infection or dissemination of the virus out of the midgut (Fig. 6) between mosquitoes infected with Ancestral virus or virus passaged at 26°C for IR (100%) or DR (100%). Viral loads were also not significantly higher (*P* = .99; Table 2) throughout infection when compared to the Ancestral lineage, with the highest load averaging at 1 × 10^7^ viral copies per microliters of supernatant. Average viral load during dissemination was similar for the two treatments and not significantly different despite the higher midgut load for Ancestral virus. Transmission rates did not significantly differ (*P* = .72) between the two. However, there was some observed variability, though not significant (*P* = .99) between the treatments with mosquitoes infected with the Ancestral line having a higher prevalence of virus-infected saliva over the time course (3 dpi = 5%, 6 dpi = 15%, 9 dpi = 100%, 12 dpi = 75%) compared to mosquitoes infected with the virus passaged at 26°C (3 dpi = 0%, 6 dpi = 35%, 9 dpi = 55%, 12 dpi = 50%). Prevalence of virus in the mosquito saliva was higher during the later end of infection (9 and 12 dpi) for both temperature lineages (Fig. 6).

**Table 2.** Performance of passaged DENV-2 lines in tissues. Select post hoc comparisons (not all) for mosquito tissue viral loads of serially passaged viruses reared at different temperatures with adjusted *P*-values.

Passage history in mosquito rearing temperature	Midgut	Carcass	Saliva
Ancestral in 26°C vs. 37°C passaged in 26°C	.017	.047	.016
26°C passaged in 26°C vs. 37°C passaged in 26°C	.0057	<.0001*	.022
Ancestral in 26°C vs. 26°C passaged in 26°C	.99	.075	.72
37°C passaged in 26°C vs. 37°C passaged in 32°C	.99	>.99	>.99

Mosquitoes infected with virus passaged at 37°C and reared at 26°C showed significantly lower infection rates in the midgut when compared to Ancestral infected mosquitoes (*P* = .017, Tukey’s post-hoc comparison; Table 2) and 26°C passaged virus in mosquitoes reared at 26°C (*P* = .0057). Viral load for midgut infection peaked at 1 × 10^4^ early in the surveyed timepoints at 3 dpi and remained significantly low thereafter until 12 dpi ranging from 1 × 10 to 10^2^ (Fig. 5). Established infections disseminated out of the midgut into the carcass (rest of the body) at a significantly slower and unsteady rate (DR at 3 dpi= 35%, 6 dpi = 75%, 9 dpi= 35%, 12 dpi = 70%; Fig. 6) compared to mosquitoes infected with the Ancestral (*P* = .047) and 26°C passaged (*P* < .0001) lines at the same temperature. Viral load during dissemination varied by day inconsistently and the highest peak load of 1 × 10^4^ occurred on the 6th day postinfection. Overall viral dissemination decayed significantly from the peak of 10^4^ and resulted in an ending load of 10. For transmission rates, only one carcass we tested from the twenty infected mosquitoes progressed to this stage likely due to the poor level of viral dissemination seen at 12 dpi (Fig. 5)

In summary, we found mosquitoes infected with Ancestral and passaged virus at 26°C had significantly higher vector competence when compared to viruses passaged at 37°C in the same condition. The ability to not effectively escape the midgut and disseminate was likely due to interactions with the midgut escape barrier. We find this pattern to be similar to what was seen *in vitro* when comparing the replicative fitness of passaged lines (Fig. 3).

## 4. Discussion

We performed *in vitro* passaging of DENV-2 in C6/36 mosquito cells for 10 passages at both low and high temperatures. Passaging at a higher temperature reduced the virus’s replicative performance at the lower temperature in both *in vitro* and *in vivo*. Convergent evolution occurred in virus replicates passaged at 37°C with an increased rate of replication leading to a higher load and the accumulation of six nonsynonymous mutations, which likely contributed to the reductions in replicative fitness at 26°C. Our findings thus suggest a possible tradeoff between virus thermostability and replication at lower temperatures in DENV-2. Nonsynonymous changes associated with the phenotypic shift were found in the envelope and nonstructural proteins, including NS2A, NS2B, and NS4A (Murugesan and Manoharan 2020). Interestingly, very few of these mutations were found in published genomes, which may suggest their poor fitness in natural environments. In a similar study investigating the adaptive potential of West Nile virus lineages to higher temperatures, mutations were identified in the same regions, including the amino acid substitutions in the envelope and NS2A proteins [37]. Unlike our study, however, the virus passaged at higher temperatures was associated with increased replicative fitness at both low and high temperatures (Fay et al. 2021).

The membrane envelope glycoprotein protein (E) of DENV is a significant determinant of virion assembly and entry (Y. Li et al. 2021). DENV depends on the low pH-mediated conformational changes in the E protein, which mediates the fusion of viral lipid membrane with host endosomal membranes—delivering the viral genome into the cytosol (Kuhn et al. 2002, Murugesan and Manoharan 2020). DENV (E) protein only has two glycosylation sites at residues N67 and N153 (Fontes-Garfias et al. 2017). Glycosylation occurs only on Asparagine in an N-X-S/T sequence, and so the N153S mutation that occurred in the 37°C passaged treatment (Line 6) would have prevented glycosylation. Glycosylation affects secondary protein processing and is critical in protein structure, function, and stability (Y. Yap et al. 2017, Li et al. 2021). Glycosylation also affects the three-dimensional structure of proteins. At the cellular level, sugar structures modulate functions that regulate cell–cell–substrate communication, host cell recognition, and adhesion (Yap et al. 2017). Mutagenesis studies have shown that a mutation at the N153 site reduced viral replication in hamster kidney cells (BHK) and C6/36 cells but not in mosquitoes infected intrathoracically (Bryant et al., 2007). Furthermore, loss of the N67 in the E protein has also been shown to affect the ability of DENV-2 to produce infectious virions in mammalian cells. However, this pattern is not present in mosquito cells (Bryant et al., 2007; Mondotte et al. 2007). Additionally, DENV2 mutant viruses that lack the N153-glycans due to single-point mutations display higher pH thresholds, suggesting that the altered fusion activity results from the instability of the E dimers (Guirakhoo et al. 1993, Yap et al. 2017). We suggest that future studies investigate further how temperature may affect the stability and structure of role N-linked glycosylation sites- N67 and N153, potentially affecting virus infection dynamics at the population level.

Nonstructural proteins play a role in RNA replication and in virion evasion of the host immune response through enzymatic activity and protein–protein interactions (Heaton et al., 2010). The nonstructural protein mutations may be more critical in thermal adaptation than the E gene mutations, given their high rate of convergent occurrence across replicates. Unlike the E gene mutations, however, there is a poorer understanding of how such mutations directly affect thermal stability or virus success. We saw a mutation at the amino acid site 136 T in the NS2A region. Based on the published topology, NS2A resides in the ER lumen similarly to prM/E and NS1 (Xie et al. 2013). However, without a known structure, we can only hypothesize that NS2A is involved in virus replication or assembly and may interact with increased temperature (Xie et al. 2013). There were two mutations in the NS2B region, one at the 113 and 114 sites. Our mutations lie in the C-terminal region of NS2B and interact with the NS3 promoter. The NS3 promoter requires NS2B for its protease activity (Luo et al. 2008; Yildiz et al., n.d.). The mutation seen here likely enhances or mediates protease activity required for polyprotein cleavage (Constant et al., 2018; Niyomrattanakit et al. 2004). One of our two mutations appears at a conserved residue and potentially has a significant role in protease activity. The AA change from Isoleucine to Threonine might enhance flexibility for the NS2B and NS3 interface, providing efficient cleavage at higher temperatures. For the NS4A mutation at AA site 93, the Tyrosine to Cysteine mutation was not a conserved residue. We predict that the Cysteine could be forming a disulfide bond with another protein or interacting membranes based on recent work describing NS4A structure (Xie et al. 2013, Zou et al. 2015).

Since the mutations we report occurred only at the higher temperature and resulted in similar patterns of reduced DENV loads in cell culture and mosquitoes, our results suggest that virus adaptation may be less contingent upon host-specific adaptation and more about temperature-specific adaptation. From arbovirus thermodynamic models, we know that there is a potential for tradeoff between thermostability and replication. All viruses, including dengue, undergo reversible fluctuations in solution called “breathing” (Kuhn et al., 2015; Lewis et al., 1998). Every dengue virus serotype/strain consequently displays a unique breathing profile dependent upon the primary sequence and stability of its tertiary and quaternary folds (Lim et al., 2017; Venkatakrishnan et al. 2024). In several dengue serotypes, the variable breathing has been demonstrated to large-scale expansion in virus structures when switched from a vector environment and human host temperature (37°C) (Fibriansah et al. 2013). Rearing at higher temperatures may select beneficial mutations that improve viral thermostability, giving the viruses an advantage in thermally stressful conditions (Singhal et al. 2017, Gale 2019). At the same time, these same mutations can cause reduced replication rates. The relative benefit of replication or enhanced thermostability becomes highly context-dependent (Domingo and Holland 1997, Singhal et al. 2017, Gale 2019). At higher temperatures, dengue expands from a particle with a smooth surface to a rough expanded particle, exposing more surfaces for receptor recognition but at the same time exposing cryptic epitopes for immune recognition. The evolutionary rates of these mutations may influence the fitness landscapes in which arboviruses like DENV will circulate (Vasilakis et al. 2009, Ciota and Kramer 2010, Choudhury et al. 2014).

Several factors may have affected our study design and should be considered in future studies. Cell lines derived from mosquito hosts provide a powerful tool for investigating host–virus interactions at the cellular level. Regardless, *in vitro* systems remain a poor representative of the vector (Walker et al. 2014). With a selection regime, it is possible for genetic bottlenecks or drift within a (relatively) small population leading to a decrease in viral fitness. Alternatively, it has been suggested that genetic bottlenecks could lead to increased fitness of the virus due to the removal of defective viruses or increased effectiveness of selection (Miyashita and Kishino 2010, Zwart and Elena 2015). Given the scale of the experimental design and poor DENV plaquing success, we utilized qRT-PCR for virus quantification. This method is known to overestimate live viruses, given the quantification of noninfectious viruses that may be produced, especially at higher temperatures. Future studies should focus on the role of cycling, given the natural constraints of both host and vector on virus evolution (Weaver 2006, Ciota and Kramer 2010, Forrester et al. 2014), using sequential passaging in the different hosts (Weaver and Barrett 2004). Such alternate passaging may lead to specialized viruses and alternate fitness gains that vary by temperature. In addition, mutated viruses could be passaged back at standard temperature conditions (26°C) to understand the potential for reversions. Recent studies in Alphaviruses found serial passaging in either mice or *A. aegypti* mosquitoes led to host-specific specialization, but that alternating passaging between the two put a break on such fitness gains (Coffey et al., 2008). The effect of daily diurnal temperature fluctuations on DENV adaptive potential should also be explored (Lambrechts et al. 2011, Carrington et al. 2013). Daily diurnal fluctuations can not only significantly affect mosquito development parameters but also influence the outcome of DENV infection, having been shown to increase infectivity in the mosquito (Ma et al. 2015, Mordecai et al. 2019). In this study, using one low and one high static temperature, we could not assess the role of temperature fluctuations in DENV evolution and adaptation.

## Supplementary Material

veaf016_Supp

## Data Availability

The data that support the findings of this study are available in Figshare at https://figshare.com/s/8a844a43e4493bee2f03.
