# Supplementary material for: Evolution and adaptation of dengue virus in response to high-temperature passaging in mosquito cells
Source: Virus Evol. 2025 Apr 24;11(1):veaf016. doi: 10.1093/ve/veaf016 (PMC12054504; doi:10.1093/ve/veaf016)
Supplement: veaf016_Supp [file veaf016_supp.zip › suppl_data/WareGilmore_Supplementary Information.pdf]

Supplemental Information

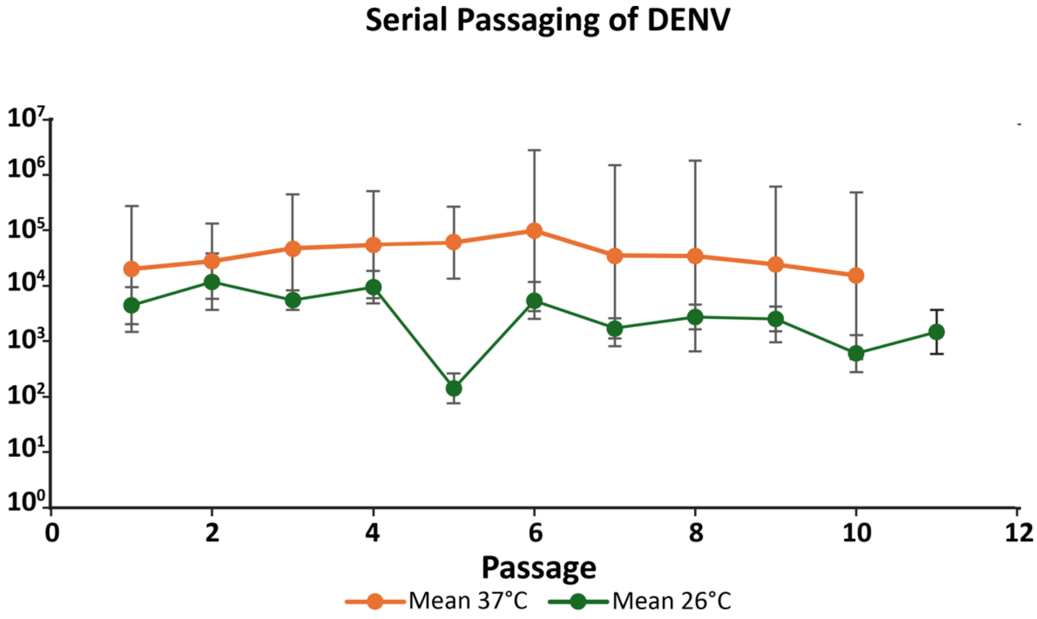

SI Figure 1. Average Viral loads during serial passaging of DENV-2 at a high and low temperature in C6/36 cells. Sequential passaging of an Ancestral DENV isolate in C6/36 mosquito cells at 26 °C (5 replicate Lines) and 37°C (7 replicate Lines). Individual points denote Log_{10} transformed viral genome copies as an average for Lines at every passage. Virus was passaged every three days, and each day virus was collected to access replication dynamics. Each point is the grand mean of viral load from within day, across days, and across Lines.

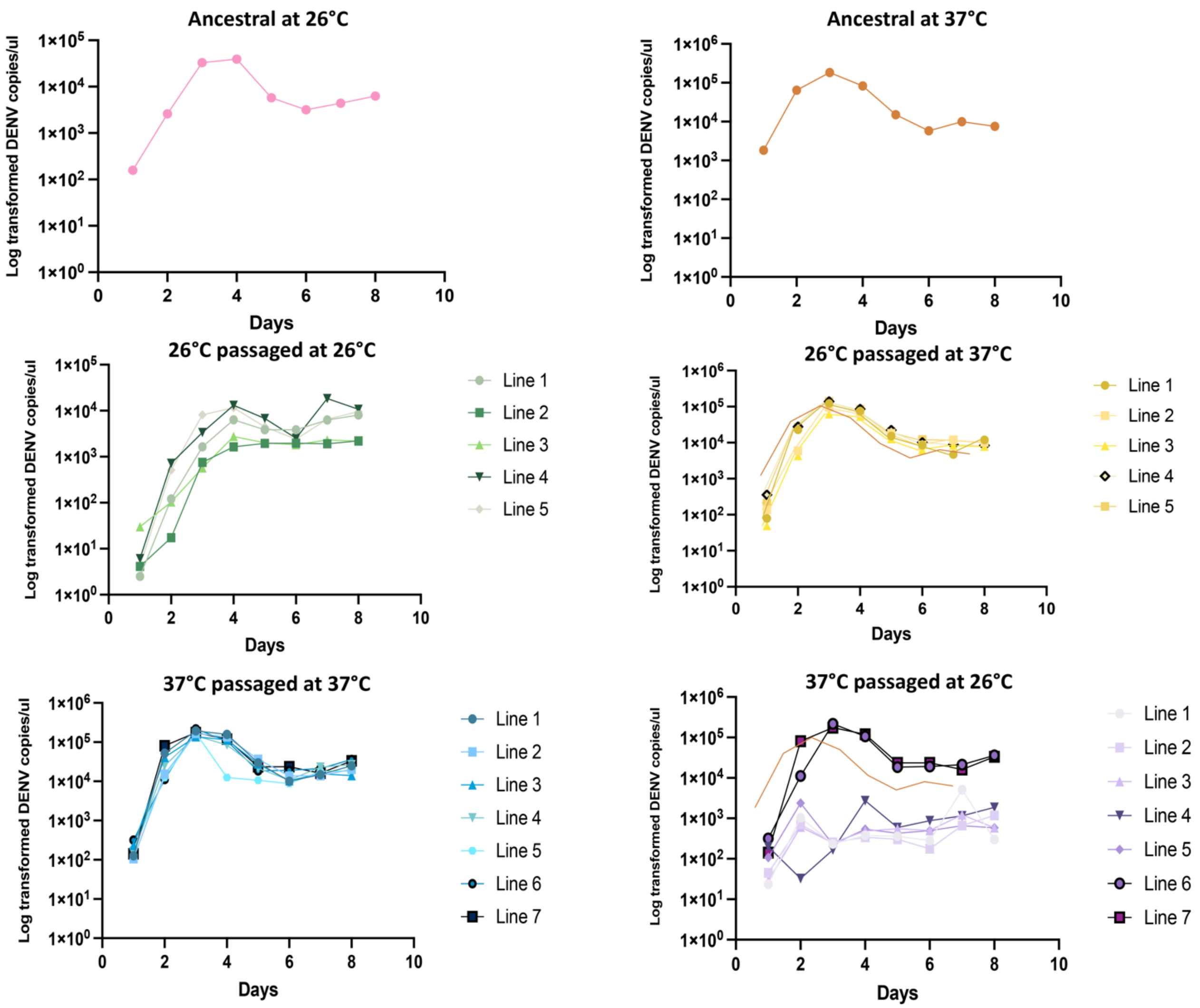

SI Figure 2. Daily viral concentrations for replicative fitness treatments with all replicates. The effect of passaging history and experimental temperature on daily viral loads across 8 days. Points represent the mean of the viral load of three collection times within a day.

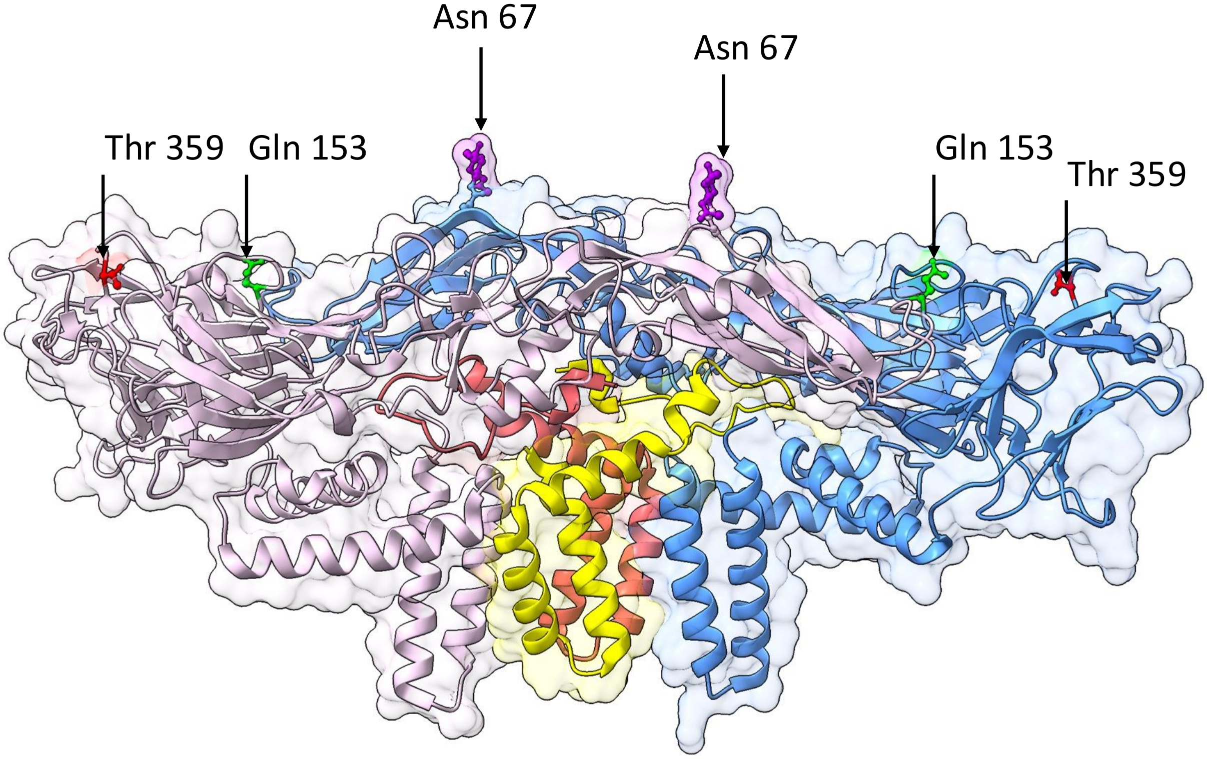

SI Figure 3. Mutation of N153 in E gene leads to the loss of glycosylation without affecting the overall architecture of M and E proteins on the virion. Surface-shaded top view of the dimeric structure of M and E heterodimers derived from the cryoEM structure of mature DENV-2 N153Q mutant from PDB ID: 8Y3G is shown with E monomers (pink and blue); M monomers (yellow and brown). The glycosylation site on Asn 67 with NAG is shown in the ball and stick model (purple). The mutation (N153Q) of Gln 153 is indicated in green. Residue Thr 359 is indicated (red).

SI Table 1. Primers and probes. Primers and probes for dengue virus strain (ET300) serotype 2 (Genbank EF440433)
targeting the 3' UTR – Poly gene.

Direction		Melting Point
Forward	5'-AAGGACTAGAGGTTAGAGGAGACCC-3'	54ºC
Reverse	5'CGTTCTGTGCCTGGAATGATG-3'	58ºC
Probe: FAM	5'-AACAGCATATTGACGCTGGGAGAGACCAGA-BHQ1-3'	

SI Table 2. Effect of passaging history on replicative fitness. Tukey's post hoc comparisons for the following fitness tests: Ancestral to 26°C, Ancestral to 37°C, 37°C passaged to 26°C, 37°C passaged to 37°C, 26°C passaged to 37°C, and 26°C passaged to 26°C

**Evolved state temperature vs. temperature during fitness assay**	**Adjusted *p*-value**
26°C to 26°C vs. 26°C to 37°C	<.00001*
26°C to 26°C vs. 37°C to 26°C	0.0016*
26°C to 26°C vs. 37°C to 37°C	<0.0001
26°C to 26°C vs. Ancestral to 37°C	0.0012*
26°C to 26°C vs. Ancestral to 26°C	0.62
26°C to 37°C vs. 37°C to 26°C	<0.0001*
26°C to 37°C vs. 37°C to 37°C	0.49
26°C to 37°C vs. Ancestral to 37°C	0.97
26°C to 37°C vs. Ancestral to 26°C	0.58
37°C to 26°C vs. v37°C to 37°C	<0.0001*
37°C to 26°C vs. Ancestral to 37°C	<0.0001*
37°C to 26°C vs. Ancestral to 26°C	0.0030*
37°C to 37°C vs. Ancestral to 37°C	1.0000
37°C to 37°C vs. Ancestral to 26°C	0.096
Ancestral to 37°C vs. Ancestral to 26°C	0.42

SI Table 3. Effect of passaging history on peak viral load. The time of maximum viral load reached at each treatment and replicate for *in vitro* all-cross experiments.

Treatment	Rep	Day	Time	Log copies DENV per ul
26 TO 26	1	4	7	4.071882007
26 TO 26	2	4	7	3.598571663
26 TO 26	3	4	7	3.862131379
26 TO 26	4	7	23	4.606381365
26 TO 26	5	4	7	4.479431337
26 TO 37	1	4	7	5.321391278
26 TO 37	2	4	7	5.266701967
26 TO 37	3	4	7	5.186391216
26 TO 37	4	4	7	5.380934463
26 TO 37	5	4	7	5.369957607
37 TO 26	5	2	15	3.808885867
37 TO 26	4	4	7	3.896526217
37 TO 26	6	4	7	3.017033339
37 TO 26	7	6	23	3.155943018
37 TO 26	1	7	23	4.146128036
37 TO 26	3	7	23	3.390935107
37 TO 26	2	8	7	3.336059278
37 TO 37	2	3	23	5.519040039
37 TO 37	4	3	23	5.445292769
37 TO 37	5	3	23	5.550961752
37 TO 37	6	3	23	5.576110894
37 TO 37	1	4	7	5.651278014
37 TO 37	3	4	7	5.490239485
37 TO 37	7	4	7	5.520090328
Ancestral 26 TO 37	1	3	23	5.42357352
Ancestral 26 TO 26	1	4	7	5.059941888

SI Table 4. ANOVA Table. Summary of Two-way ANOVAs for viral load of DENV-2 passaged at alternate temperatures in *Ae. aegypti* tissues

**Midgut**	**Sum of square**	***df***	**MS**	**F (DFn, DFd)**	***P*-Value**
**Between treatments**	8268880890007	5	1653776178001	F (5, 474) = 11.62	*P*<0.0001

**Carcass**	**Sum of square**	***df***	**MS**	**F (DFn, DFd**	***P*-Value**
**Between treatments**	2.294e+014	5	45881643482828	F (5, 474) = 18.18	*P*<0.0001

**Saliva**	**Sum of square**	***df***	**MS**	**F (DFn, DFd)**	***P*-Value**
**Between treatments**	40994486852	5	8198897370	F (5, 474) = 3.43	*P*=0.0047

SI Table 5. Parameters used to describe vector competence for DENV-2 passaged at 26°C and 37°C. This table reports Infection rate (IR), dissemination rate (DR), transmission (TR) rate, and for DENV positive individuals.

*n = total number of challenged mosquitoes, *A*= number with virus present in midgut,
B= number with virus present in carcass, *C* = number with virus present in saliva,
Dpi = days post infection

	**% IR (A/N) (Midgut)**	**% DR (B/N) (Carcass)**	**% TR (C/N) (Saliva)**
**Ancestral at 26°C**			
3 dpi	100 % (20/20)	100 % (20/20)	5 % (1/20)
6 dpi	100 % (20/20)	100 % (20/20)	15 % (3/20)
9 dpi	100 % (20/20)	100 % (20/20)	100 % (20/20)
12 dpi	100 % (20/20)	100 % (20/20)	80% (16/20)
**Ancestral at 32°C**			
3 dpi	100 % (20/20)	100 % (20/20)	75% (15/20)
6 dpi	100 % (20/20)	100 % (20/20)	75% (15/20)
9 dpi	100 % (20/20)	100 % (20/20)	80% (16/20)
12 dpi	100 % (20/20)	100 % (20/20)	95% (15/20)
**26°C Passaged at 26°C**			
3 dpi	100 % (20/20)	100 % (20/20)	0 % (0/20)
6 dpi	100 % (20/20)	100 % (20/20)	60 % (12/20)
9 dpi	100 % (20/20)	100 % (20/20)	60 % (12/20)
12 dpi	100 % (20/20)	100 % (20/20)	55 % (11/20)
**26°C Passaged at 32°C**			
3 dpi	100 % (20/20)	100 % (20/20)	20% (4/20)
6 dpi	100 % (20/20)	100 % (20/20)	100 % (20/20)
9 dpi	100 % (20/20)	100 % (20/20)	75% (15/20)
12 dpi	100 % (20/20)	100 % (20/20)	100 % (20/20)
**37°C Passaged at 32°C**			
3 dpi	95 % (19/20)	75 % (15/20)	0 % (0/20)
6 dpi	100% (20/20)	90 % (18/20	35 % (7/20)
9 dpi	100 % (20/20)	100 % (20/20)	55 % (11/20)
12 dpi	100 % (20/20)	100 % (20/20)	50 % (10/20)
**37°C Passaged 26°C**			
3 dpi	50 % 10/20)	35 % (7/20)	5 % (1/20)
6 dpi	40 % (8/20)	75 % (15/20)	0 % (0/20)
9 dpi	35 % (7/20)	35 % (7/20)	0 % (0/20)
12 dpi	5 % (1/20)	70 % (14/20)	0% (0/20)
